# Pedaling Asymmetry Reflected by Bilateral EMG Complexity in Chronic Stroke

**DOI:** 10.3390/e26070538

**Published:** 2024-06-23

**Authors:** Shi-Chun Bao, Rui Sun, Raymond Kai-Yu Tong

**Affiliations:** 1 National Innovation Center for Advanced Medical Devices, Shenzhen 518110, China; 2 Department of Biomedical Engineering, The Chinese University of Hong Kong, Hong Kong SAR, China; 3 Paul C. Lauterbur Research Center for Biomedical Imaging, Shenzhen Institute of Advanced Technology, Chinese Academy of Sciences, Shenzhen 518055, China; 4 Department of Rehabilitation Sciences, The Hong Kong Polytechnic University, Hong Kong SAR, China; rui-eliza.sun@polyu.edu.hk

**Keywords:** EMG, stroke, fuzzy approximate entropy, pedaling asymmetry, complexity

## Abstract

This study examines pedaling asymmetry using the electromyogram (EMG) complexity of six bilateral lower limb muscles for chronic stroke survivors. Fifteen unilateral chronic stroke and twelve healthy participants joined passive and volitional recumbent pedaling tasks using a self-modified stationary bike with a constant speed of 25 revolutions per minute. The fuzzy approximate entropy (fApEn) was adopted in EMG complexity estimation. EMG complexity values of stroke participants during pedaling were smaller than those of healthy participants (*p* = 0.002). For chronic stroke participants, the complexity of paretic limbs was smaller than that of non-paretic limbs during the passive pedaling task (*p* = 0.005). Additionally, there was a significant correlation between clinical scores and the paretic EMG complexity during passive pedaling (*p* = 0.022, *p* = 0.028), indicating that the paretic EMG complexity during passive movement might serve as an indicator of stroke motor function status. This study suggests that EMG complexity is an appropriate quantitative tool for measuring neuromuscular characteristics in lower limb dynamic movement tasks for chronic stroke survivors.

## 1. Introduction

Stroke is a cerebro-cardiovascular disease that induces death and severe disability throughout the world, and millions of people suffer from stroke every year in the USA [1]. Most stroke survivors experience lower limb motor impairments which are tough to recover from. Such reduced functional mobility and independence severely decrease their quality of life and induce huge burdens to their family and the whole society [2]. Though many rehabilitation interventions have been employed for lower limb motor recovery, such as robotics [3], brain–computer interface [4], and electrical stimulation [5], the rehabilitation efficiency remains limited. A more in-depth understanding of motor deficiencies in stroke patients might contribute to more efficient rehabilitation designs. In addition to conventional clinical assessment scores, stroke-related motor impairment is correlated with structural and functional metrics derived from neurophysiological and neuroimaging techniques, such as magnetic resonance imaging, transcranial magnetic stimulation, electromyography, and human motion measurement and analysis [6,7].

Among these techniques, surface electromyography (sEMG) provides electrophysiological measurements of muscles and can be used as neurorehabilitation control signals and assessment tools of neuromuscular functions [8]. Though EMG has been widely applied in stroke-related neurorehabilitation, EMG-based lower limb asymmetry assessment was not extensively applied when compared with other techniques based on motion capture, reaction force, or pressure measurements [9,10]. Lower limb movements require complex sensorimotor integrations with multiple spatial and temporal scales. The high nonregularity and nonlinear nature of myoelectric signals cannot be adequately described by linear measures like EMG amplitude [11]. Instead, nonlinear measures have been employed in EMG signal processing, such as entropy analysis, considering the degree of randomness/nonregularity in time series, and the increase in entropy values indicates the increase in complexity and the decrease in regularity [12,13]. Entropy measures have been applied to assess gait symmetry using stance force or stride intervals [14], and the high consistency and accuracy of the entropy-based algorithm made it a suitable tool for measuring lower limb movement asymmetry. Therefore, complexity-based techniques could also be applicable in unveiling lower limb movement symmetry using sEMG signals.

Approximate entropy (ApEn) and its derivatives have been applied in biomedical signal processing with a high accuracy of neural signals [15]. More recently, fuzzy approximate entropy (fApEn) and fuzzy sample entropy (fSampEn) algorithms have improved the robustness and consistency of ApEn and SampEn by incorporating Zadeh’s fuzzy sets [11]. An earlier study showed that elbow EMG fApEn values of stroke survivors were smaller than those of healthy controls, and the number and firing rate of active motor units were both reduced after stroke impairment using simulated EMG signals [16]. This reduction is attributed to impaired descending central drive due to central nervous system damage and subsequent neurogenic and myopathic changes in paretic muscles [17]. Additionally, an increased motoneuron number might contribute to motor function recovery after robot-based motor training in chronic stroke patients [18]. It was also reported that the complexity-based techniques were more robust than the conventional root mean square method [19]. Subsequently, entropy-based complexity measures could serve as examination tools for neuromuscular changes after stroke [20]. Further, the EMG complexity could potentially be used as a new indicator for spasticity for stroke patients [21]. The easy applicability of EMG complexity measures might facilitate extensive clinical applications.

However, it is limited to the functional states of stroke survivors, and some patients have difficulty in walking or performing complex lower limb tasks. Upright stationary pedaling training is an alternative to walking tasks for stroke survivors, which shares similar muscular control strategies [22]. Further, both volitional and passive motor tasks might provide information regarding the functional states of the muscular system [23]. Human pedaling requires inter-limb motor coordination, but pedaling symmetry is severely influenced after stroke [24]. Pedaling asymmetry might be caused by reduced muscular activities and impaired interlimb coordination [25]. Scrutinizing pedaling asymmetry after stroke might facilitate our understanding of neuromuscular control and the recovery process. As far as we know, there has been no complexity-based pedaling asymmetry analysis; it might provide new perspectives to examine neuromuscular systems and serve as potential tools for neurological assessment, postoperative follow-up, and motor rehabilitation [26].

This study was proposed to examine the utility of sEMG complexity in measuring the pedaling asymmetry in chronic stroke and healthy participants. It was hypothesized that task-specific pedaling asymmetry could be investigated by bilateral sEMG complexity in stroke survivors. Additionally, sEMG complexity might be related to the residual motor functions of chronic stroke participants.

## 2. Materials and Methods

### 2.1. Participants

This study was conducted on fifteen first-ever unilateral chronic stroke survivors with moderate lower limb motor impairments. Stroke survivors were excluded if they had (1) any additional medical or psychological condition that would affect their ability to comply with the study protocol, e.g., a significant orthopedic or chronic pain condition or major post-stroke depression; or (2) severe hip, knee, or ankle contracture or spasticity that would preclude the passive range of motion of the leg. Well-trained examiners conducted the clinical assessment, and demographic characteristics are illustrated in Table 1 (9 females, 6 males; age: 59.0 ± 10.5 years; time since stroke onset: 5.8 ± 3.6 years; Fugl-Meyer Assessment Lower Extremity (FMA-LE) motor score: 22.6 ± 4.0, range: 16–28, full mark: 34; Berg Balance Scale (BBS) score, 51.3 ± 4.9, range: 41–55, full mark: 56; paretic side: 7 right; ischemic: 10; all right-leg dominated before stroke onset), and stroke lesion information was acquired from hospital records. Moreover, twelve male young healthy volunteers (age: 25.4 ± 5.1 years, all right-leg dominant) were involved in the experiment. All participants declared no lower limb muscular disorders or injuries for the last two years. Before experiments, all participants signed an informed consent approved by the Joint Chinese University of Hong Kong-New Territories East Cluster Clinical Research Ethics Committee.

### 2.2. Study Paradigm

**Figure 1:**
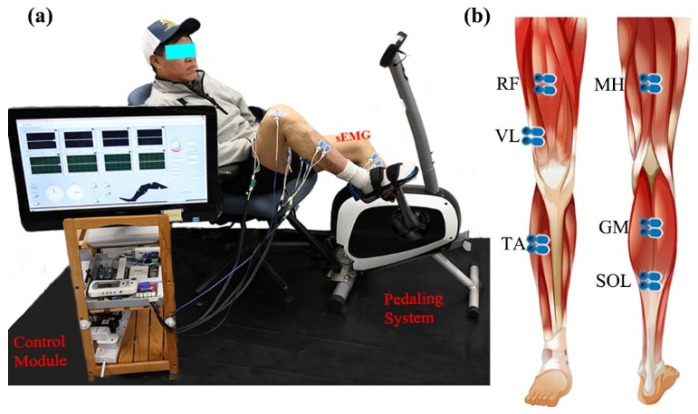
Surface EMG and the stationary pedaling system. (**a**) The general structure of the measurement system, including stationary bike, control module, and experimental position. (**b**) The placement of surface EMG electrodes.

Bilateral lower limb EMG signals were collected during different recumbent pedaling tasks. The experimental system included an upright stationary bike with a tunable armchair (Figure 1a). The detailed designs could be found in previous studies [27,28]. The pedaling system was powered by a step motor with an angle measurement tool (UIM241 controller, UIRobot Inc., Shanghai, China) and a planetary gear. Torque sensors (HLT132, Hualiteng Inc., Shenzhen, China) were installed on the pedal cranks to measure inter-crank torque symmetry. Bilateral surface EMG signals were recorded with an NI PCIe-6320 DAQ (National Instruments, Austin, TX, USA) and an amplifier (gain of 1000 at 3 dB; input impedance: 10 GΩ; recording bandwidth: 0–380 Hz) [29]. Six representative lower limb muscles were selected, including medial hamstring (MH, knee flexion), rectus femoris (RF, knee extension), vastus lateralis (VL, knee extension), gastrocnemius (GM, ankle plantarflexion), soleus (SOL, ankle plantarflexion), and tibialis anterior (TA, ankle dorsiflexion), see Figure 1b. Surface EMG electrodes (BlueSensor N, Ambu, Denmark) were placed according to SENIAM requirements with a center-to-center distance of 2 cm [30]. The quality of sEMG signals was carefully examined. Further, a Labview-based graphical user interface was utilized for pedaling control and signal collection with a 1 kHz sample rate.

Two recumbent pedaling tasks were conducted on the same day with a 10 min inter-task break. Each task lasted 12 min, with passive pedaling tasks arranged before volitional tasks to minimize the carry-over effects and muscle fatigue [31]. For volitional tasks, stroke survivors should pedal voluntarily with their paretic limbs, while healthy volunteers should use their left limbs, the non-paretic limbs (stroke survivors), or right limbs just relaxed and following the pedal movement. In passive pedaling tasks, all participants were required to relax and follow the pedal movement as required, and no additional efforts were allowed for both limbs for all the subjects [32,33]. Lab experimenters gave verbal instructions to help participants pedal as expected based on real-time muscular responses and torque values displayed in the Labview interface. Additionally, pre-experiment adjustments were arranged before the experiments to ensure smooth pedaling and effective muscular activation. Participants started each task with their left leg or paretic leg at the top dead center (TDC) position with a crank angle of 0/360°, where participants had maximum knee flexion. The largest range of motion of knee extension was maintained at 140–150°. The Labview module kept the pedaling speed stable, with the first and last minutes serving as the warm-up or cool-down periods at 15 revolutions per minute (RPM). Only the middle 10 min in the 25 RPM pedaling period was selected for later EMG analysis.

### 2.3. Data Analysis

EMG signals were processed using the Matlab 2018b signal processing toolbox. Then, a 30–350 Hz four-order Butterworth filter and a 50 Hz notch filter were used to remove the powerline interference and unwanted frequencies [34]. The EMG, torque, and angle signals were synchronized, and only the 25 RPM pedaling periods were selected. Afterward, EMG signals were segmented into epochs with the length of pedaling cycles (2.4 s), starting from the TDC position. EMG epochs with abnormal fluctuation (more than three times the average fluctuation amplitude) were discarded. An average of 242.8 ± 16.6 EMG epochs for healthy participants and an average of 246.3 ± 19.6 epochs for stroke participants were kept in each task, respectively. Subsequently, EMG signals were used for complexity and conventional EMG metrics estimation. Conventional EMG metrics include both EMG envelope values and the muscular activation period. The mean linear envelope was obtained from a 5 Hz low-pass-filtered full-wave rectified EMG signal. The durations where EMG envelope values were larger than 20% of the maximum envelope values were taken as the muscular activation period [35].

### 2.4. Fuzzy Approximate Entropy and Fuzzy Sample Entropy

We adopted two fuzzy entropy measures to calculate pedaling sEMG complexity: fApEn and fSampEn [36]. The fApEn algorithm characterizes signal complexity by integrating the fuzzy membership function in conventional ApEn measures to define the similarity of vectors [37]. For a signal of N-sample time series $N\left(x_{1},x_{2},\ldots ,x_{N}\right)$, fApEn is defined as the conditional probability that two vectors remain similar when the vector dimension increases from *m* to *m* + 1.

Firstly, we construct a set of vectors in a delayed m-dimensional vector space, $X_{i}^{m}=\{x(i),x(i+1),\ldots ,x(i+m-1)\}-x0(i)$, (i=1,2,…,N−(m−1)τ),j≠i; here, *τ* is the time delay, x0(i) is the baseline value, and $x0(i)=\frac{1}{m}\sum_{j=0}^{m-1}x(i+j)$.

The similarity $D_{ij}^{m}(n,r)$ of two m-dimensional vectors is defined as the distance $d_{ij}^{m}$ between a pair of corresponding measurements of $X_{i}^{m}$ and $X_{j}^{m}$, $D_{ij}^{m}\left(n,r\right)=\exp\left(-{\left(\frac{d_{ij}^{m}}{r}\right)}^{n}\right)$. Where *n* is the gradient of the boundary of the exponential function with the value of 2, the symbol *r* represents a predefined tolerance value that determines the width of the fuzzy exponential function of the similarity measure.

(1) $$\begin{array}{cc} d_{ij}^{m} & =d[X_{i}^{m},X_{j}^{m}]\\ & =\underset{k\in (0,m-1)}{max}|(x(i+k)-x0(i))-(x(j+k)-x0(j))| \end{array}$$

Further, the conditional probability Φ*^m^* (*N*, *r*) is defined as the following equations:

(2) $$\Phi^{m}\left(N,r\right)={\left[N-\left(m-1\right)\tau \right]}^{-1}\sum_{i=1}^{N-\left(m-1\right)\tau }\ln\left[C_{i}^{m}\left(N,r\right)\right]$$

and

(3) $$C_{i}^{m}\left(N,r\right)=\frac{1}{N-\left(m-1\right)\tau }\cdot \sum_{j=1}^{N-\left(m-1\right)\tau }D_{ij}^{m}\left(n,r\right)$$

Finally, fApEn can be estimated as

(4) $$fApEn\left(m,r,N\right)=\Phi^{m}\left(N,r\right)-\Phi^{m+1}\left(N,r\right)$$

The fSampEn is an extension to SampEn and has a similar form to fApEn [15], but fSampEn does not consider the self-match of every vector like fApEn. fSampEn might have better entropy performance in short time series [36]. The conditional probability Φ*^m^* (*N*, *r*) is estimated as the following equation [12], where

(5) $$\Phi^{m}(N,r)=[N-m\tau {]}^{-1}\sum_{j=1,j\neq i}^{N-m\tau }D_{ij}^{m}(n,r)$$

Similarly, we have

(6) $$\Phi^{m+1}(N,r)=[N-m\tau {]}^{-1}\sum_{j=1,j\neq i}^{N-m\tau }D_{ij}^{m+1}(n,r)$$

Further, fSampEn is defined as

(7) $$fSampEn(m,r,N)=ln(\Phi^{m}(N,r))-ln(\Phi^{m+1}(N,r))$$

### 2.5. EMG Simulation and Fuzzy Entropy Parameter Selection

The entropy of the whole EMG time series was further obtained by averaging the entropy of individual N-sample segments. Regarding complexity estimation parameters, three parameters (*m*, *r*, and *N*) should be determined beforehand. We choose vector dimension *m* = 2 for EMG signal processing as reported previously [37]. Moreover, the tolerance *r* value is in the range of 0.1–0.5 and EMG segment length *N* is in the range of 100–500 [15]. We utilized simulated pedaling EMG signals to determine appropriate *r* and *N* values in complexity estimation. Further, the simulated pedaling EMG signals were used for characterizing critical factors influencing pedaling EMG complexity, such as the number of recruited motor units, the mean firing rate, and noise levels, and the result might facilitate interpretations of EMG pedaling asymmetry analysis in stroke and healthy participants.

EMG signals of typical muscles during pedaling were simulated with a volume conductor model [38]. Specifically, surface EMG contraction patterns were achieved by integration of multi-layer motor unit action potentials which could be measured with the summation of single fiber action potentials. The single fiber action potential *ϕ* (*x*, *y**,** z*) is determined as

(8) $$\varphi (x,y,z)=\frac{\sigma_{I}}{4\pi \sqrt{\sigma_{Y}\sigma_{Z}}}\int_{s}ds\int_{-\infty }^{\infty }\frac{\partial \phi (z)}{\partial z}\frac{\partial (r^{-1})}{\partial z}dz$$

where $\phi (z)=96z^{3}e^{-z}-90$ is the intracellular potential of the action potential, *z* is the axial direction, and *s* is the fiber section. *σ_I_* is the intracellular conductivity, and *σ_Z_* and *σ_Y_* are the muscle axial and radial conductivity, respectively. Further, we could obtain motor unit action potential (MUAP) by summing the single fiber action potential as $V_{mu}=\sum_{n=1}^{N_{f}}\varphi_{n}$, where the timing of motor units could be expressed as $t_{st,n}=n\Delta t_{int}=\frac{n}{f_{r}N_{mu}}$, and *n* =1,2,…,$N_{mu}$, *f_r_* is the mean firing rate. Then, the MUAP trains could be expressed as

(9) $$V_{\mathrm{muts}\;}=\{V_{\mathrm{mut},1},V_{\mathrm{mut},2},\cdots ,V_{\mathrm{mut},N_{\mathrm{mu}\;}}\}$$

where the firing rates fulfill Poisson distribution [39]. Furthermore, the simulated surface EMG signal during pedaling is obtained by summing all motor unit action potential trains in the selected time range. Further, 50 dB Gaussian random noise is added to mimic real measurement. Surface EMG contraction and relaxation patterns during pedaling cycles were specially considered by setting the firing rate range and the maximum contraction period [40], where the firing rate of the steady contraction period is around 8–40 Hz and the firing rate of the maximum contraction period is 70–90 Hz [31]. For different muscles, the pre-defined muscle activation patterns are estimated according to classical pedaling EMG profiles, including the range of activation ranges and the maximum contraction periods [41]. Moreover, the numbers of recruited motor units are set according to several related studies [42]. To summarize, all pedaling EMG simulation parameters are included in Table 2.

The simulated sEMG signals (10 cycles, duration 24 s) during pedaling were used to determine suitable entropy estimation parameters. We compared the performance of two entropy measures by changing the segment length N and tolerance r; the simulated EMG time series were divided into N-sample segments for entropy estimation. The segment length N was set in the 100–500 range with a step of 50, and the tolerance r value was in the 0.15–0.45 range with a step of 0.05. Further, suitable parameters were chosen based on the overall complexity performance in different muscles. Subsequently, based on selected entropy estimation parameters, we explored potential entropy changes with EMG simulation settings by tuning the number of recruited motor units, the mean firing rate, and the signal-to-noise ratio (SNR). Additionally, the actual pedaling EMG signals were divided into N-point segments with a step of 100 points overlapping, and the mean entropy values were calculated for each muscle, side, task, and participant.

### 2.6. Statistical Analysis

All statistical analyses were conducted using IBM SPSS Statistics 22 (IBM, Armonk, NY, USA). Shapiro–Wilk’s test was used to examine data distribution conditions. Values out of the 95% confidence interval (mean ± two standard deviations, SD) were corrected using the mean values [43]. Three-way analysis of variance (ANOVA) (task × sides × channels: 2 × 2 × 6) was conducted to explore the main effects of pedaling tasks, limb sides, EMG channels, and their interactions on bilateral lower limb EMG complexity and conventional EMG metrics for healthy and stroke participants. The *p*-value was adjusted using the Greenhouse–Geisser Epsilon correction in the case of sphericity violation (Mauchly’s test). Additionally, a paired *t*-test was used with Bonferroni’s correction. Further, due to different sample sizes and inhomogeneous data distributions between stroke and healthy participants, a nonparametric Mann–Whitney U test was adopted to determine the EMG metrics differences between stroke and healthy participants. Pearson correlation was utilized to investigate the relationship between clinical scores and EMG entropy. The significance level was set at *p* < 0.05.

## 3. Results

### 3.1. Simulated EMG and Fuzzy Entropy Parameter Selection

**Figure 2:**
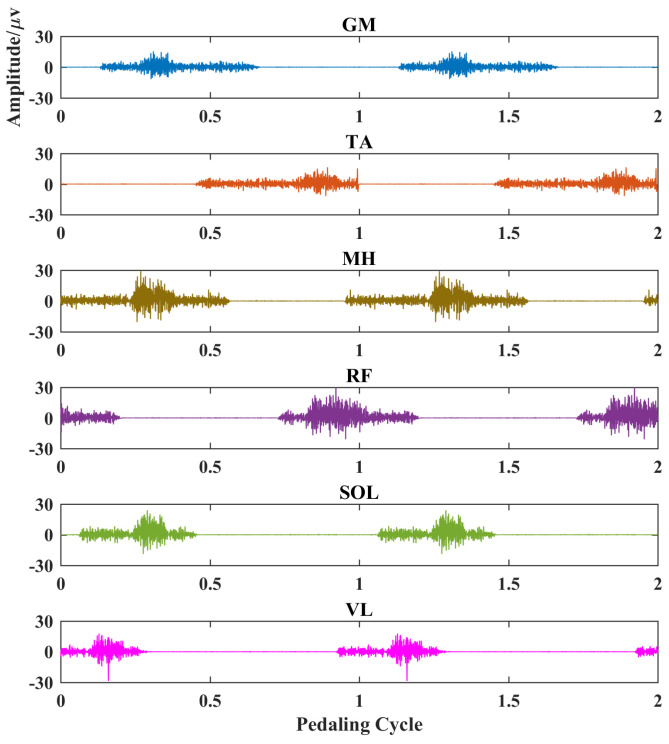
EMG simulation results for six typical muscles in two pedaling cycles. Each subplot represents a different muscle. X-axis, pedaling cycle; Y-axis, simulated EMG amplitude. GM: gastrocnemius medialis, TA: tibialis anterior, MH: medial hamstring, RF: rectus femoris, SOL: soleus, VL: vastus lateralis.

**Figure 3:**
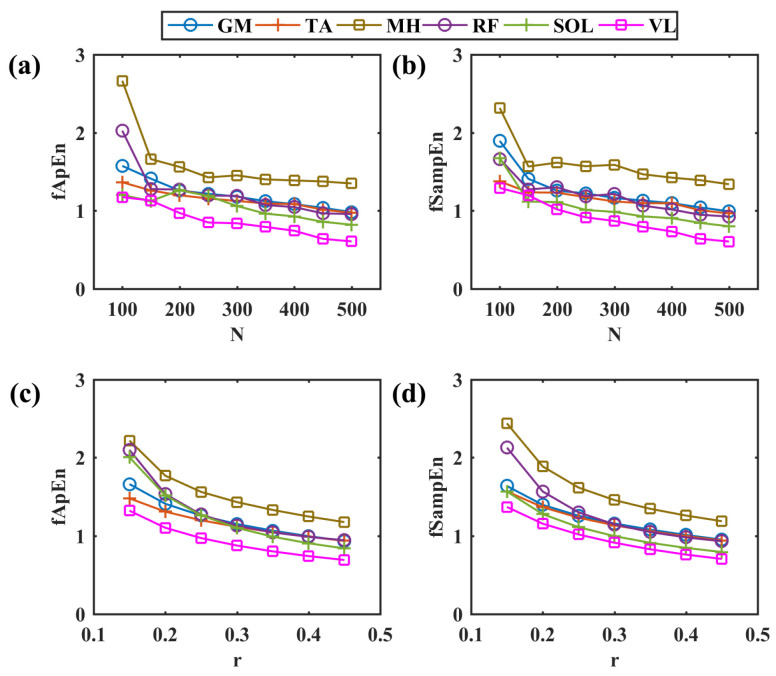
Comparisons among different entropy parameters. (**a**,**b**) The change in fApEn and fSampEn with the increase in different segment lengths in a step of 50, *r* = 0.25. (**c**,**d**) The change in fApEn and fSampEn with the increase in different r values in a step of 0.05, *N* = 200. GM: gastrocnemius medialis, TA: tibialis anterior, MH: medial hamstring, RF: rectus femoris, SOL: soleus, VL: vastus lateralis.

The simulated myoelectric responses of six muscles in two pedaling cycles are shown in Figure 2, and typical muscle contraction and relaxation patterns in pedaling cycles are illustrated. Based on simulated EMG signals, Figure 3 presents comparisons between two different complexity measures. For a fixed tolerance *r* = 0.25, Figure 3a,b shows a decreasing trend of entropy with an increase in *N* values from 100 to 500, and *N* = 200 is enough to stabilize the entropy values. Similarly, Figure 3c,d demonstrates a similar decreasing trend with fixed segment length *N* = 200. The two fuzzy entropy measures exhibit comparable performance for simulated EMG signals. As fApEn was more widely used in other studies, we chose fApEn for subsequent analysis with complexity parameters of *N* = 200 and *r* = 0.25.

**Figure 4:**
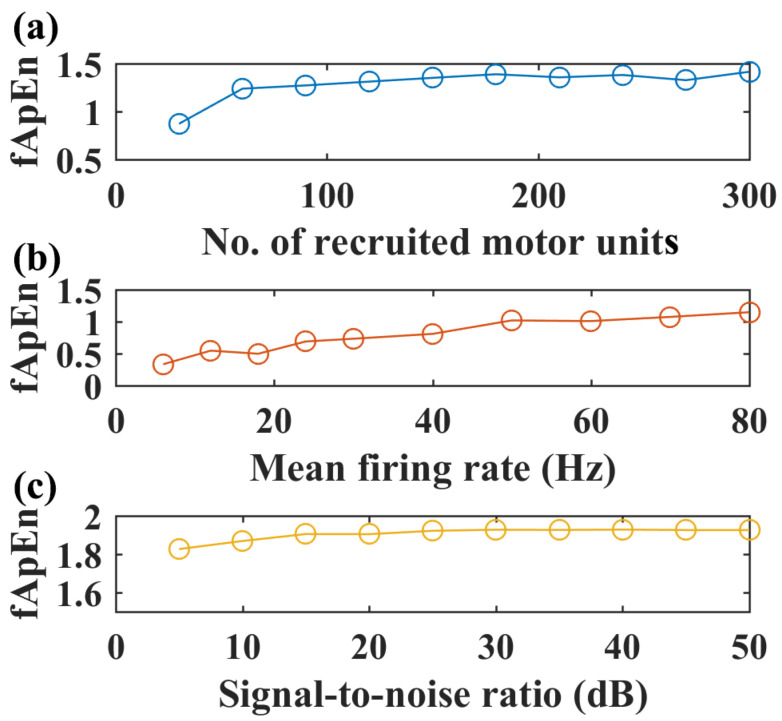
Simulated EMG complexity with different recruited motor units, motor unit firing rates, and SNR levels, *N* = 200, *r* = 0.25. (**a**) The complexity of simulated sEMG of the GM muscle with an increased number of recruited motor units, firing rate: 12 Hz, SNR level: 50 dB. (**b**) Complexity of simulated EMG signals with increased motor unit firing rate, number of recruited motor units: 150, SNR level: 50 dB. (**c**) Complexity of simulated EMG signals with increased signal-to-noise level, firing rate: 12 Hz, number of recruited motor units: 150.

With the selected fApEn parameters, the changes in fuzzy entropy values with different recruited motor units, motor unit firing rates, and SNR levels are shown for the simulated EMG of the GM muscle in Figure 4. An increased number of recruited motor units can increase fApEn values, but fApEn remains stable with more than 150 motor units (Figure 4a). Likewise, fApEn values increase with the mean firing rate, but such an increasing trend is not typical with more than a 50 Hz firing rate (Figure 4b). Additionally, simulated signals with more than a 20 dB signal-to-noise ratio might be enough to have stable fApEn estimations, as shown in Figure 4c.

### 3.2. Comparisons of Kinematics and Conventional EMG Metrics in Pedaling

The mean inter-crank torque values during two pedaling tasks were compared for both healthy and chronic stroke participants. In healthy volunteers, significantly larger mean torque values (*p* < 0.001) were found in volitional pedaling (4.06 ± 2.30 N·m) compared with passive pedaling (−0.05 ± 0.77 N·m). Similarly, for stroke survivors, significantly larger mean torque values (*p* < 0.001) were observed in volitional pedaling tasks (5.04 ± 1.52 N·m) compared with passive pedaling (2.27 ± 2.09 N·m). Smaller torque values were reported in healthy participants than in chronic stroke participants (*p* = 0.001, ANOVA with Welch correction for inhomogeneous variances).

**Figure 5:**
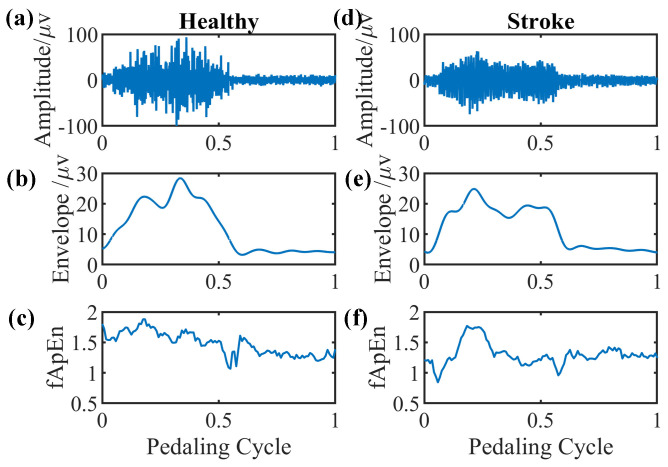
Representative GM muscle EMG signal and corresponding complexity in a single active pedaling cycle. (**a**–**c**) The actual left-side EMG of GM muscle during active pedaling, the EMG envelope, and the complexity for healthy participant 11. (**d**–**f**) The actual paretic-side EMG of GM muscle during active pedaling, the EMG envelope, and the complexity for stroke participant 4.

**Figure 6:**
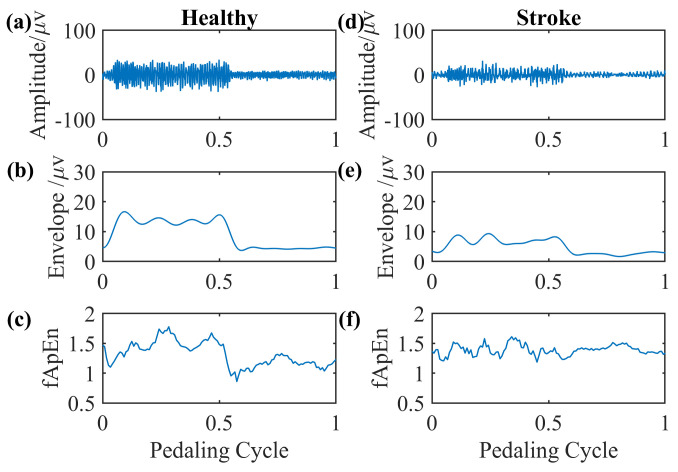
Representative GM muscle EMG signal and corresponding complexity in a single passive pedaling cycle. (**a**–**c**) The actual left-side EMG of the GM muscle during passive pedaling, the EMG envelope, and the complexity for healthy participant 11. (**d**–**f**) The actual paretic-side EMG of the GM muscle during passive pedaling, the EMG envelope, and the complexity for stroke participant 4.

We select EMG signals of the left-side GM muscle during volitional pedaling as an example, and the corresponding EMG envelope and complexity for healthy participant 11 are presented in Figure 5a–c. The simulated GM EMG signals in Figure 2 exhibit similar patterns to real EMG signals here. For stroke survivor 4, we found similar paretic-side EMG signals in volitional pedaling, as shown in Figure 5d–f. Additionally, we select EMG signals of the left-side GM muscle during passive pedaling as an example, and the corresponding EMG envelope and complexity for healthy participant 11 are presented in Figure 6a–c. For stroke survivor 4, we found similar paretic-side EMG signals in passive pedaling, as shown in Figure 6d–f.

Overall, larger EMG envelope values and longer activation periods were observed in healthy volunteers compared to chronic stroke survivors (*p* = 0.002, *p* < 0.001, Mann–Whitney U test). Specifically, larger EMG envelope values and longer activation periods were found in healthy volunteers compared to stroke patients for passive pedaling tasks, (*p* = 0.003, *p* < 0.001, Mann–Whitney U test). For volitional pedaling tasks, longer activation periods were reported in healthy participants compared to chronic stroke survivors (*p* = 0.002, *p* < 0.001, Mann–Whitney U test). Regarding the comparison between two sides using conventional EMG metrics in stroke subjects, for volitional pedaling tasks, smaller EMG envelope values were observed on the volitional paretic side compared to the non-paretic side (*p* < 0.001). However, for the passive pedaling task, smaller EMG envelope values and shorter activation duration of the volitional paretic side were observed when compared with those of the non-paretic side (both *p* < 0.001).

### 3.3. Factors Influencing fApEn in Healthy Participants

A three-way ANOVA was conducted to explore the impact of pedaling tasks, muscle sides, and sEMG channels on bilateral pedaling EMG complexity for healthy and stroke participants, as measured by fApEn. All fApEn values fulfilled normality distribution as examined by the Shapiro–Wilk’s test (*p* > 0.05). For healthy volunteers, the interaction effect of channels*tasks*sides was not statistically significant (*p* > 0.05). In contrast, the interaction effects of channels*tasks, channels*sides, and tasks*sides are significant (*p* < 0.05), and the main effects of channels and tasks are significant (*p* < 0.001). The three-way ANOVA results are presented in Table 3 on the left side. The overall fApEn values during volitional pedaling were smaller than those during passive pedaling (*p* < 0.001, Bonferroni corrected).

**Figure 7:**
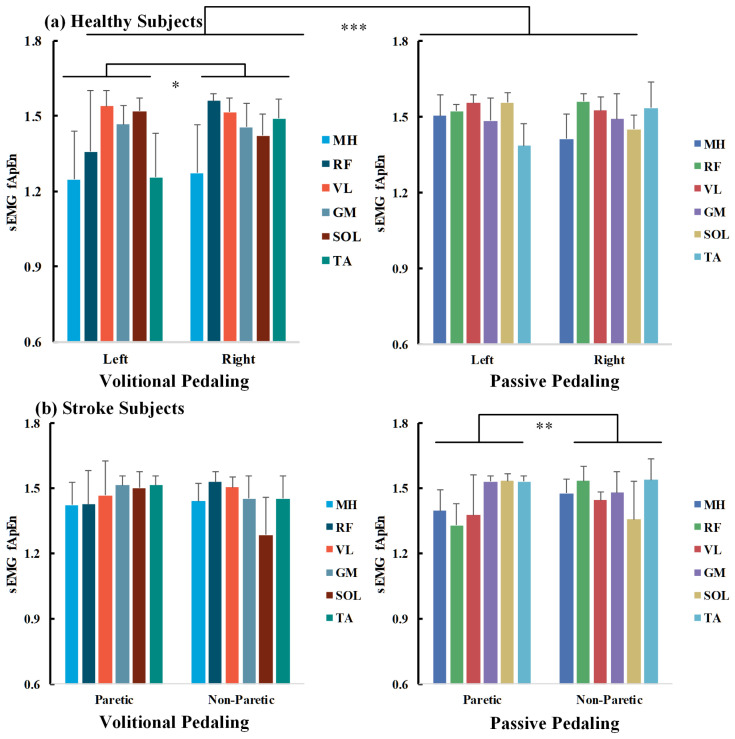
fApEn comparisons across pedaling tasks, sides, and muscle channels for healthy (**a**) and chronic stroke survivors (**b**). Average across survivors, half error-bar = ±1 SD, ***, *p* < 0.001, **, *p* < 0.01, *, *p* < 0.05.

For individual EMG channels, separate two-way ANOVA found significant main effects of sessions, especially for the MH, RF, and TA channels (*p* < 0.001, *p* = 0.029, and *p* = 0.024). For volitional pedaling, two-way ANOVA demonstrated the significant main effects of sides and channels (*F*(1,132) = 6.499, *p* = 0.012, partial *η*^2^ = 0.047, *F*(5,132) = 12.727, *p* < 0.001, partial *η*^2^ = 0.325, respectively), and interaction effects of sides*channels (*F*(5,132) = 6.341, *p* < 0.001, partial *η*^2^ = 0.194). The pairwise between-limb comparison showed that the volitional EMG fApEn (left side) was significantly smaller than that of the right side (*p* = 0.012, Bonferroni corrected). Further one-way ANOVA showed channel-specific fApEn patterns in different recumbent pedaling tasks and sides (*p* < 0.01), suggesting that different muscles responded differently during pedaling tasks. For example, the post hoc test showed that MH has smaller fApEn than other channels, see Figure 7a’s left part. Whereas for the passive pedaling task of healthy volunteers, no significant difference between the two sides was reported in the two-way ANOVA (*p* > 0.05), showing that the bilateral EMG complexity was similar in the passive pedaling task for healthy participants, see Figure 7a’s right part.

### 3.4. Factors Influencing fApEn in Chronic Stroke Participants

As for chronic stroke survivors, a significant interaction effect of tasks*sides was found (*F*(1,336)= 5.188, *p* = 0.023), as shown in Table 3 on the right side. For volitional pedaling, a two-way ANOVA illustrated the significant main effect of channels (*F*(5,168) = 5.631, *p* < 0.001, partial *η*^2^ = 0.144) and interaction effect of channels*sides (*F*(5,168) = 8.564, *p* < 0.001, partial *η*^2^ = 0.203). Post hoc analysis showed no significant difference between paretic and non-paretic lower limb muscles (*p* > 0.05), see Figure 7b’s left part. Regarding the passive pedaling task, two-way ANOVA illustrated a significant main effect of sides (*F*(1,168) = 8.045, *p* = 0.005, partial *η*^2^= 0.046) and interaction effect of channels*sides (*F*(5,168) = 16.275, *p* < 0.001, partial *η*^2^ = 0.326). The post hoc tests showed a significant difference between the paretic and non-paretic limbs (*p* = 0.005, Bonferroni corrected), and the overall complexity of the paretic side is smaller than that of the non-paretic side, see Figure 7b’s right part. No significant channel-level fApEn difference was observed in passive pedaling.

### 3.5. fApEn and Its Clinical Implications

Considering the fApEn difference between chronic stroke and healthy participants, the overall muscular EMG complexity of stroke survivors was significantly smaller than that of healthy volunteers (*p* = 0.002, Mann–Whitney U test). As the right limbs of healthy and non-paretic limbs of stroke participants were required not to pedal volitionally, the mean sEMG fApEn values across different muscles showed similar trends, as indicated in Figure 7. Specifically, for passive pedaling tasks, the paretic EMG complexity in stroke participants was smaller than that of the volitional left side in healthy participants (*p* < 0.001, Mann–Whitney U test). However, for volitional pedaling tasks, we did not find a significant difference between stroke and healthy participants.

**Figure 8:**
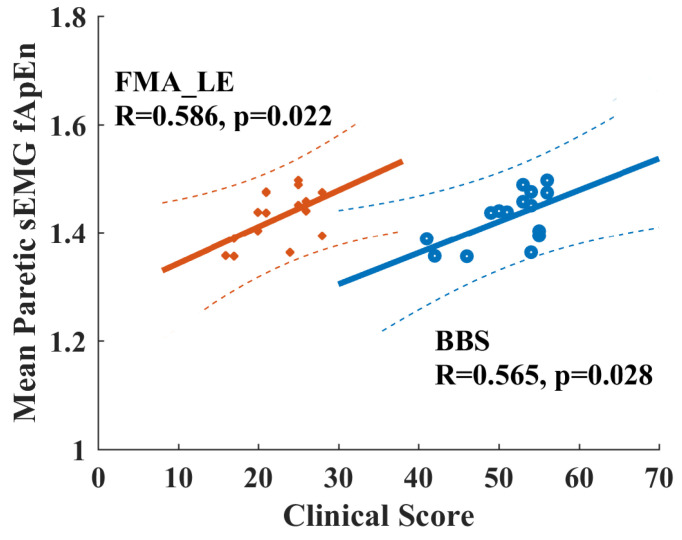
Positive correlation between clinical scores and EMG fApEn of the paretic leg during passive pedaling in chronic stroke survivors. Orange points and lines are for the relationship between FMA-LE and fApEn. Blue points and lines are for the relationship between BBS and fApEn. Dash line, 95% prediction interval. FAM-LE: Fugl-Meyer Assessment Lower Extremity; BBS: Berg Balance Scale.

When checking the potential relationship between fApEn values with clinical scores for chronic stroke survivors, none of the EMG fApEn values during volitional pedaling are significantly correlated with FMA-LE or BBS scores (*p* > 0.05). However, there is a significant Pearson correlation between the mean paretic fApEn values during passive pedaling and FMA-LE/BBS score (FMA-LE vs. mean paretic fApEn, *R* = 0.565, *p* = 0.028, BBS vs. mean paretic fApEn, *R* = 0.586, *p* = 0.022, see Figure 8). Specifically, the paretic VL muscle during passive pedaling showed significant correlations with FMA-LE (*R* = 0.658, *p* = 0.008) and BBS scores (*R* = 0.522, *p* = 0.046), and the VL muscle exhibited the highest R-value. Such positive correlations implied that paretic EMG fApEn might serve as a potential indicator for measuring residual motor functions in chronic stroke survivors.

## 4. Discussion

The current study investigated pedaling asymmetry using bilateral EMG complexity in healthy and chronic stroke participants. Complexity analysis was analyzed with the fuzzy approximate entropy technique, and we found that the EMG complexity values of stroke participants were smaller than those of healthy participants. Moreover, the bilateral pedaling symmetry could be task-dependent for both stroke and healthy participants. For chronic stroke survivors, the overall complexity of paretic limbs was smaller than that of non-paretic limbs in passive pedaling. However, there was no statistical difference in inter-limb complexity for the volitional pedaling task. Additionally, the paretic EMG complexity might serve as a potential indicator of stroke motor functional status.

For healthy participants, they can conduct pedaling tasks as required. As shown in Figure 7a, no significant inter-limb fApEn difference was observed in passive pedaling for healthy controls, and the torque values were nearly zero. These findings implied symmetrical bilateral muscular activations in passive movement for healthy participants [28]. For volitional pedaling, the entropy values of the volitional left side were smaller than that of the right side. The conventional EMG metrics analysis demonstrated a shorter activation period for the volitional left side than the no-efforts right side, which might influence the EMG entropy properties. For passive pedaling tasks, it is typical that most participants can only reduce the EMG during passive when compared with volitional pedaling but not to zero. The passive pedaling process may require processes such as the alteration of descending corticospinal drive, inhibition of reflexes, and inhibition of pattern-generating circuits [33]. Involuntary muscle activities are associated with complicated MUAP properties [43], which may explain smaller complexity on the volitional side. Furthermore, continuous passive wrist manipulation was demonstrated to introduce nonlinear contributions to the corticomuscular control process [44]. Likewise, nonlinearity could be introduced during passive pedaling tasks; thus, smaller fApEn values might be expected for volitional-side EMG. Simple conventional EMG metrics might be limited in robustness and consistency in the dynamic motor tasks [25], and the robustness of EMG fApEn measures to noise could be demonstrated in Figure 4c. The conventional and nonlinear entropy measures might supplement each other. Indeed, the relationship between linear and nonlinear measures should be interpreted carefully [45,46].

However, chronic stroke survivors with motor impairments cannot move normally like healthy participants; the paretic limbs cannot relax and follow the pedal movement like the non-paretic limbs as required. Thus, the inter-limb torque values during passive pedaling were not zero (2.27 ± 2.09 N·m), while the torque values of healthy subjects were nearly zero during passive pedaling tasks (−0.05 ± 0.77 N·m), which might explain the different entropy performance, especially in lower limb muscles when compared with healthy controls. Smaller sEMG complexity was observed in paretic limbs compared to non-paretic limbs, suggesting a reduced number of recruited motor units and decreased firing rate of active motor units [47,48]. These results are further supported by our EMG simulation results in Figure 4, where fApEn values increase with the number of recruited motor units and firing rate. Conventional EMG metrics showed smaller envelope values and shorter activation periods for the paretic legs than the non-paretic legs, which were consistent with fuzzy entropy findings.

Regarding the volitional paretic pedaling of stroke survivors, we found no significant difference in EMG complexity between the two limbs, suggesting that paretic volitional efforts could facilitate muscular activations when compared with non-paretic involuntary legs [18]. However, the mean EMG complexity across paretic muscles during passive pedaling showed significant positive correlations with clinical scores, as shown for the vastus lateralis (VL) in Figure 8. Such findings demonstrated that complexity measures in passive dynamic tasks might serve as potential quantitative indicators for stroke motor recovery, which was supported by the fact that upper limb grip strength was correlated with complexity analysis [49,50]. Definitely, more efforts are required to translate such fApEn findings into clinical applications, and the functional role of complexity analysis with conventional EMG metrics should be clearly demonstrated in future studies. Additionally, Hu et al. measured the dynamic stretch reflex threshold (DSRT) by passively moving elbows with a spasticity measurement system, and spectrum entropy-based DSRT measures were correlated with modified Ashworth scale scores for stroke patients with elbow flexor spasticity [51], thus their results also supported current findings. In this study, we selected six typical muscles for analysis, three proximal muscles for knee movement, and another three distal muscles for ankle movement. The overall complexity of the paretic side was smaller than that of the non-paretic side during passive pedaling tasks for stroke survivors, especially the proximal muscles (MH, RF, and VL) for knee movement. It was reported that motor units in the proximal muscles tended to degenerate more than distal muscles following chronic stroke [52], potentially explaining the special role of the paretic VL muscle in measuring clinical status. Future studies with fewer EMG channels might facilitate practical applications, but muscle-specific patterns in different motor tasks should be carefully interpreted. Additionally, current EMG measurements could not investigate the number of recruited motor units and firing rates, and subsequent studies with high-density EMG might resolve the limitations of this study.

Considering other limitations of this study, it should be acknowledged that no gender- and age-matched healthy participants participated in this study. Future studies with more age-matched healthy participants might be needed to determine muscular entropy changes and facilitate the understanding of the underlying muscular activation patterns in different motor tasks for stroke and healthy subjects. Additionally, more stroke participants with a similar stroke onset history or homogeneous cerebro-cardiovascular pathogeny could improve the reliability of further studies. From the aspect of experimental designs, future studies should consider the functional roles of the unaffected legs during lower limb motor tasks. In this study, constant-speed pedaling was applied for both passive and volitional tasks in both stroke and healthy participants; future studies might utilize self-paced motor tasks to facilitate stroke rehabilitation. Moreover, future muscle synergy analysis could explain more about the neuromotor control patterns and the neuromusculoskeletal properties after chronic stroke [53]. Further, the inter-limb coordination [25] and cortico-spinal drive patterns [54] were not discussed; future studies might shed light on neuromotor control mechanisms during pedaling together with cortical measurement tools.

Pedaling asymmetry characteristics are suitable assessment tools for measuring pathological properties, especially for people with severe lower limb motor impairment, and such entropy-based pedaling asymmetry measures might provide a reliable complement to clinical motor evaluations. In the current study, we found a significant correlation between clinical scores and mean paretic fApEn during passive pedaling tasks. The fApEn measures focus more on the internal randomness of EMG signals instead of the conventional EMG amplitude, and no extra maximum voluntary contraction tasks are required, making it convenient for potential clinical applications [16]. Beyond this, such pedaling asymmetry measures might be extended to the clinical evaluation of neuromuscular improvements after therapeutic or rehabilitation efforts. These characteristics might provide critical insights into more reliable treatment or rehabilitation interventions.

## 5. Conclusions

In summary, this study proposed EMG complexity as a tool for measuring pedaling asymmetry in chronic stroke survivors. The paretic EMG complexity was smaller than that of the non-paretic side during passive pedaling tasks, and such paretic EMG complexity might imply possible clinical indicators for stroke participants. EMG complexity exploration may help characterize post-stroke neuromuscular properties in dynamic movement tasks, and such a tool might facilitate later stroke rehabilitation applications.

## Figures and Tables

**Table 1:** Characteristics of the chronic participants.

Participant	Age	Gender	TSS	Type	FMA-LE	BBS	Affected Side	Lesion Location
1	59	F	7.3	H	24	54	R	Cortical L
2	59	F	6.3	H	20	51	R	Subcortical L
3	60	F	7.4	I	25	53	R	Subcortical L
4	54	F	14.8	I	26	53	L	Cortical R
5	69	M	10.4	I	26	50	L	Cortical R
6	37	M	4.6	I	21	49	R	Cortical L
7	72	F	5.8	I	25	56	R	Cortical L
8	63	M	2.2	I	17	41	L	Cortical R
9	57	M	2.3	I	21	54	L	Cortical R
10	72	F	4.2	I	25	54	L	Cortical R
11	61	M	9.0	I	28	55	L	Subcortical R
12	61	M	3.4	H	28	56	R	Subcortical L
13	65	F	3.9	H	16	42	L	Cortical R
14	60	F	2.9	I	20	55	R	Subcortical L
15	36	F	2.1	H	17	46	R	Cortical L

F = Female; M = Male; H = Hemorrhage; I = Ischemic; R = Right; L = Left; FMA-LE, Fugl-Meyer Assessment Lower Extremity, only motor part, full: 34; BSS, Berg Balance Scale, full: 56; TSS, time since stroke (years).

**Table 2:** Parameters for pedaling EMG simulation.

Parameters	Value
Muscle axial conductivity	*σ_Z_* = 0.328*Sm*^−1^
Muscle radial conductivity	*σ_Y_* = 0.063*Sm*^−1^
Intracellular conductivity	*σ_I_* = 1.010*Sm*^−1^
Fiber diameter	d = 55 ± 10 µm, mean ± SD; Gaussian distribution
Single fiber conduction velocity	V = 2.2 + 0.05 × (d − 25), d in µm
Muscle radius	Length: mean = 100 mm, SD = 1 mm, Gaussian distribution; position: uniform distribution
Motor unit	Circle shape; diameter: range 5–10 mm, lambda = 6 mm, Poisson distribution; position: uniform distribution
Motor unit firing rate	Range from 8–90 Hz, maximum contraction, range 70–90 Hz, steady, 8–40 Hz, lambda: 12 Hz, Poisson distribution
Pedaling cycle	2400 ms/cycle (360°)
Muscle activation pattern and No. of recruited motor unit	
Gastrocnemius medialis	N_MU = 150, range: 45–225°, max: 90–120°
Tibialis anterior	N_MU = 150, range: 160–360°, max: 280–320°
Medial hamstring	N_MU = 250, range: 340–190°, max: 80–120°
Rectus femoris	N_MU = 250, range: 260–60°, max: 290–350°
Soleus	N_MU = 200, range: 20–150°, max: 80–110°
Vastus lateralis	N_MU = 150, range: 330–80°, max: 30–60°

**Table 3:** Three-way ANOVA results for pedaling EMG complexity using fApEn.

Source	Healthy	Stroke
Channels	*F*(5,264) = 15.957, *p* < 0.001, partial *η*^2^ = 0.232	*F*(5,336) = 4.674, *p* < 0.001, partial *η*^2^ = 0.065
Tasks	*F*(1,264) = 31.034, *p* < 0.001, partial *η*^2^ = 0.105	*F*(1,336) = 0.665, *p* = 0.415
Sides	*F*(1,264) = 3.590, *p* = 0.059	*F*(1,336) = 2.366, *p* = 0.125
Channels*Tasks	*F*(5,264) = 4.566, *p* = 0.001, partial *η*^2^ = 0.080	*F*(5,336) = 2.654, *p* = 0.023, partial *η*^2^ = 0.038
Channels*Sides	*F*(5,264) = 11.711, *p* < 0.001, partial *η*^2^ = 0.182	*F*(5,336) = 22.910, *p* < 0.001, partial *η*^2^ = 0.254
Tasks*Sides	*F*(1,264) = 5.594, *p* = 0.019, partial *η*^2^ = 0.021	*F*(1,336) = 5.188, *p* = 0.023, partial *η*^2^ = 0.015
Channels*Tasks*Sides	*F*(5,264) = 1.327, *p* = 0.253	*F*(5,336) = 0.197, *p* = 0.310

## Data Availability

The data presented in this study are available on request from the corresponding author.
